# Repair of a Cracked Historic Maryan Bell by Gas Welding

**DOI:** 10.3390/ma14102504

**Published:** 2021-05-12

**Authors:** Dariusz Bartocha, Czesław Baron

**Affiliations:** Department of Foundry, Silesian University of Technology, 7 Towarowa St., 44-100 Gliwice, Poland; dariusz.bartocha@polsl.pl

**Keywords:** high-tin bronzes, microstructure, welding of bell, bell’s sound

## Abstract

In this article, the range of works connected with the repair of a historical Maryan bell from 1639 are presented. The first attempts to repair damaged bells occurred in the 1930s in Poland. However, this process was stopped because of extensive technological difficulties. Welding and soldering-welding were the basic methods. There is one difference between these two methods—connecting surfaces are melted during the welding process but only heated until the melting temperature of the material added to the connection (that is the solder) during the soldering-welding process. It was important to heat the bell to the proper temperature during welding. Uneven heating causes the enlargement of existing cracks or the appearance of new ones, or even the complete destruction of the bell. Nowadays, a method of even heating using a special heating mat has been devised. Thanks to this method it is possible to control the heating and cooling process. The most important task during the whole operation of bell welding was obtaining the original sound. During this research, the chemical composition was examined to prepare a welding rod with a suitable chemical composition. After the repair process, an analysis of the sound of the bell was conducted. It was shown that the repair of bells is possible when correct thermal parameters are used. The most highly recommended technique for repairing bells is gas welding.

## 1. Introduction

There are a lot of churches with very old, historical bells in Poland, as it has historically been a Catholic country. Unfortunately, their strength is decreasing and cracks and scratches are have appeared. These defects make them useless, because not only does the sound become worse, but reacting to the damage too late can cause the complete destruction of the bell as well.

The lifetime of the bell was determined to be 200–300 years [1] on the basis of data in the literature. After that time the probability of the bell cracking is increased. Of course, it depends on many factors, such as the frequency of bell work and the bell’s rotation on its suspension. Unless the bell is rotated, the clapper hits the same place and it may cause cracks. It is important to control the thickness of that place. If the thickness falls more than 10%, it is necessary to rotate the bell to allow it to hit another place. Constant hits on the same place also causes changes to the inner structure of the material. It becomes harder and loses strength properties. Concurrently, inner stresses increase. When stresses exceed the material strength limit, the bell will be damaged, and cracks and scratches will appear. It is hard to notice such a crack on a bell placed high in a tower, but it is possible to hear the change in the sound, which is usually much worse than the original sound.

The repair of the bell is possible up to its complete destruction. Repairing the bell is an expensive and time-consuming process. However, attempts to repair these bells are not rare, because the bell is a precious item not only thanks to its material value but also its historical and artistic value as well. Despite the avoidance of the repair of cracked bells in Poland for a long time, this problem has been considered in many other countries [2,3].

It is necessary to examine the chemical composition of the alloy used to produce the bell to repair the crack. The oldest bells are made of gunmetal, while the younger are made of bronze. Copper at a concentration of about 80% and tin with a concentration between 19% and 21% are the main components in both alloys. Gunmetal also contains zinc, lead, carbon, and iron. Trace amounts of silver and gold are also possible to observe in both alloys because of the tradition to add these elements into the liquid metal to ennoble the material [4,5,6].

**Figure 1:**
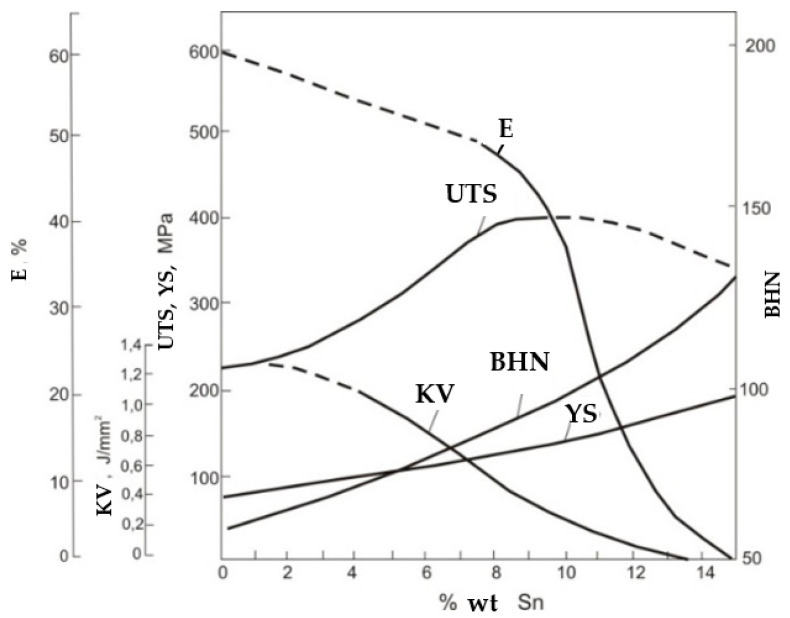
The influence of tin on chosen mechanical properties, the tin bronze hardness change dependent on tin concentration. (E—elongation, UTS—ultimate tensile strength, BHN—Brinell hardness number, KV—impact strength, YS—yield strength) [7].

**Figure 2:**
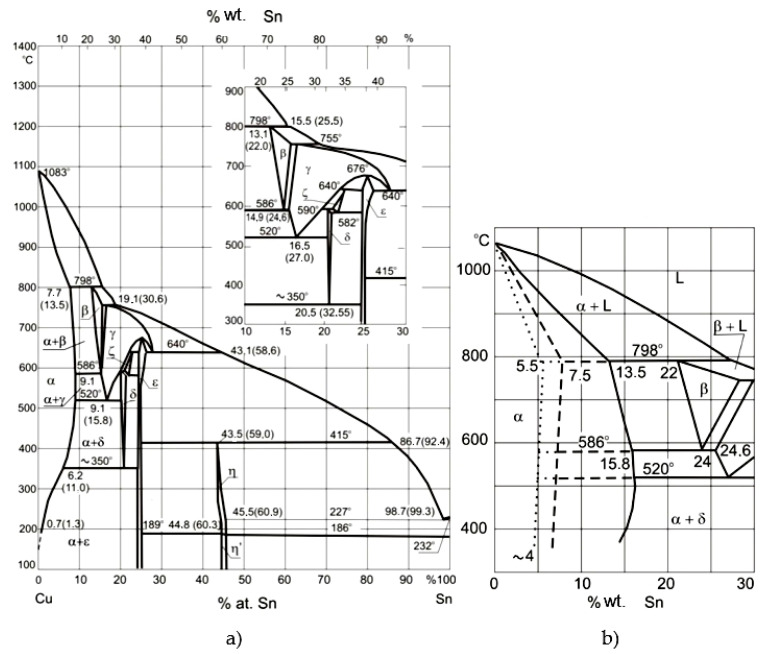
(**a**) Phase diagram Cu-Sn, (**b**) Metastable phases Cu-Sn; dashed line—casting solidified in sandy mold, dotted line—casting solidified in a metal mold [8,9].

The melting temperature of bronze is about 850–950 °C. The alloys with high tin content are characterized by great strength, but concurrently low toughness—they are very brittle (Figure 1). This, in combination with high thermal expansion and high diversity of the microstructure component properties, has a negative influence on weldability. It is proper to heat the whole bell at the adequate speed until it reaches the temperature of 350–450 °C, before welding. This allows differences in temperature between the welded place and the rest of the bell which are too large to be avoided. What’s more, it is also important to cool it slowly and evenly (the speed of the cooling process should be slower than heating). This is connected with the risk of inducing residual heat as a result of differences in the cooling rates of different parts of the structure (Figure 2a,b shows different phases according to different cooling rates). If the bell had not been heated, only the welded part would have shrunk and new cracks would have appeared. On the other hand, a cooling process which is too fast may cause new stresses, with new cracks as the result.

## 2. Historical Background

In Poland, the first attempts to repair damaged bells were observed in the 1930s. Welding and soldering-welding were the base methods. There is one difference between these two methods: connecting surfaces are melted during welding process and only heated until the melting temperature of the material added to the connection (that is solder) during the soldering-welding process. Additionally, a welding rod with a chemical composition similar to indigenous metal was used during welding process, whilst sticks made of brass alloy (known as bronzite) were used for soldering-welding.

First, it was desirable to estimate the size of the crack. A simple penetration study was conducted with the use of chalk and kerosene. Chalk was rubbed into the inner side of the bell and the external side was lubricated with kerosene. The greasy spot was used to show the range of the crack. After estimating the size of the damage, the place of repair should be properly prepared. In both cases, the method of action was similar. A groove with a v–shape was cut along the crack and metal was poured in. In this procedure the hole at the end of the crack must be remembered. Its task was to limit the increase of the crack during bell heating. An acetylene torch was used as the welding tool in both methods. Uniformity of the weld obtained was the main difference between welding and the soldering-welding process. The weld had nearly the same chemical composition as welded material during the welding process. The weld had different chemical composition to the repaired bell during the soldering-welding process, which caused worse sound.

The position heated by charcoal (in the past it was often used as a fuel) was used to heat the bell before repair. This solution was connected with uneven bell heating and cooling. It may have caused new cracks or increased the old ones as a result.

## 3. Maryan Bell Crack Repairing

The company Rduch Bells & Clocks and Foundry Department collectively made a decision to repair a cracked Maryan bell from the 17th century at the request of urban restorer in Krosno–Marta Rymar. The bell hangs in a church tower. It is the smallest bell of three cast in 1639, weighing 580 kg. It is characterized by producing a “G#” sound. The bell was cast by two bell founders, Szepan Meutel and Jerzy Olivier, for a special order from the great philanthropist Robert Wojciech Portius. The Maryan bell is one of three treated bells. On the tower there are also the Urban bell and the Jan bell. The bells are tuned to a major scale. This means that they sound happy, merry, and concurrently noble. The lack of one bell or unclear sound will cause the whole set to lose its musical value. This is why it was so important not only to repair the crack but to do it in such a way as to avoid changes to the sound. 

The most important task during welding process was to obtain the original sound. Fortunately, in 2013, during the change of the clapper, acoustic measurements were conducted. Thanks to this it was possible to obtain the sound before and after the damage to the bell (the damage occurred in 2017—exact date is unknown). This was the base for further activities. The work was divided into a few steps:

1. The first step was to take a material sample to determine the averaged chemical composition of the alloy, which was analyzed with a glow–discharge spectrometer LECO GDS500A (LECO Corporation, St. Joseph, MI, USA, 2011) (Table 1);
2. In the next step a series of welding rods with the same chemical composition were prepared on the basis of these results, which were used during bell welding process;
3. The next step was to determine the size and range of the crack, and penetration research was conducted;
4. The next step was preparing the bell for the welding process by properly bevelling the sides of the bell;
5. The next step was heating and keeping the bell at the proper temperature;
6. The next step was obtaining the required temperature to conduct welding process;
7. After welding slow cooling was conducted to avoid stresses;
8. The last step was analysis of the sound of the repaired bell.

**Figure 3:**
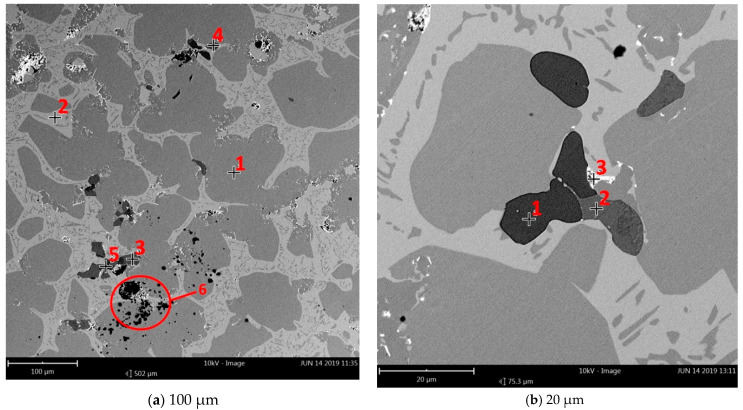
Location of the measurement points for scanning microscope examination with visible impurities and inclusions. (**a**) 1–6 measurement points for magnification 530×, (**b**) 1–3 measurement points for magnification 800×.

Accuracy in all of these activities allowed us to obtain the ideal sound from the repaired bell. The samples of material obtained were examined with the use of a spectrometer to determine averaged chemical composition (presented in Table 1). The analysis of structure was also conducted with the use of a scanning microscope to determine the distribution and size of solid and gaseous inclusions. (Figure 3a,b). The chemical composition in particular places (with visible solid and gaseous inclusions) was presented in Table 2.

Metallographic microsections showed the original structure of the bell. Unfortunately, many gaseous (Figure 3a, 6) and non–metallic inclusions were observed. A large amount of carbon (Figure 3b, 1) can indicate residue of charcoal, which was used as fuel during the melting process. Zinc inclusions were also observed (Figure 3a, 5).This negatively influenced the welding process.

**Figure 4:**
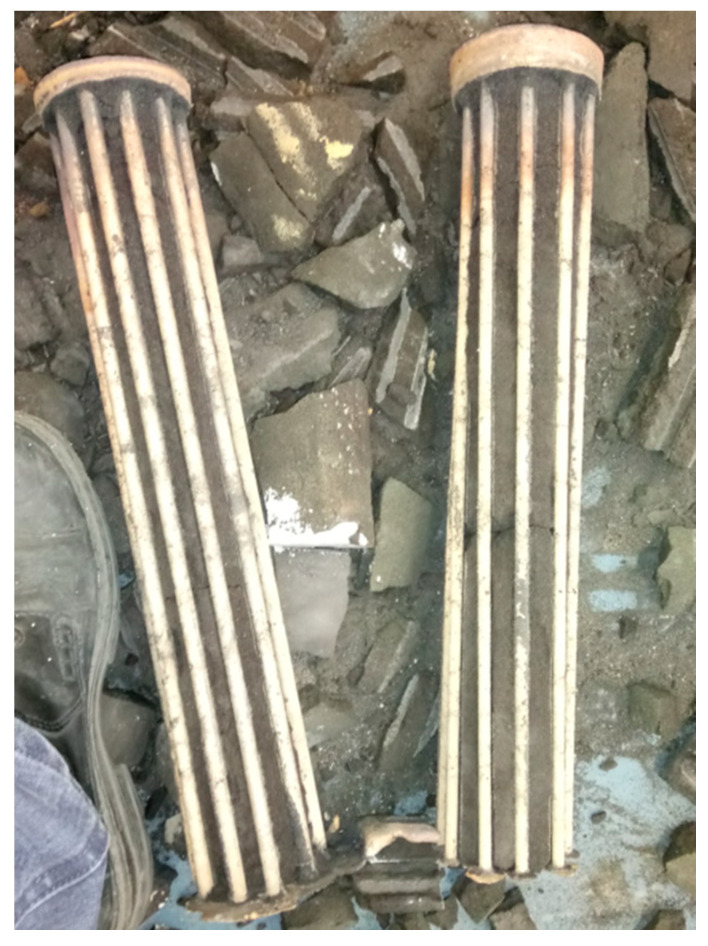
Welding rods of different lengths and diameters (the length of rods was 40 cm; the diameters were 8 mm and 6 mm).

A series of welding rods were made after chemical composition determination and consultations with the company conducting the welding process. Their composition was selected to be as compatible as possible with the examined material of the bell. A set of molds was worked out and prepared in the Foundry Department, and thanks to them welding rods of different lengths and diameters were produced (Figure 4).

**Figure 5:**
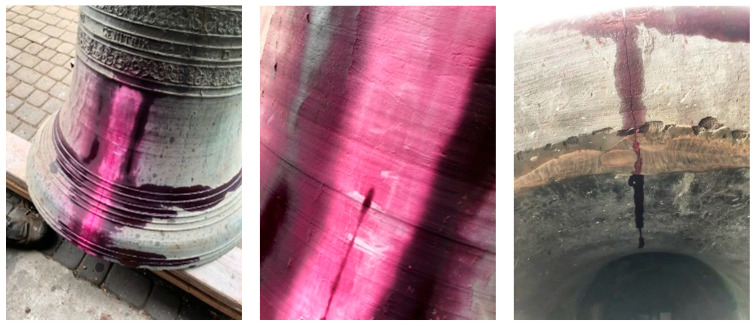
Penetration testing.

The size and range of the crack were examined with the use of penetration testing (Figure 5). Penetrator was used for this examination by covering the crack and film, which helped to determine the range of the crack.

**Figure 6:**
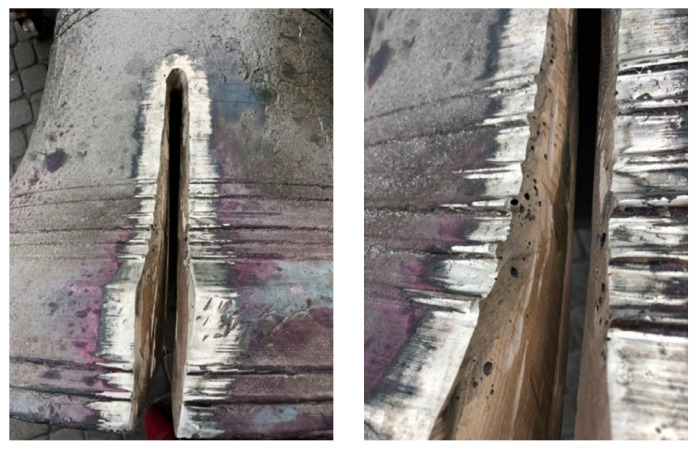
Mechanical treatment of the damaged place.

After crack range determination, mechanical treatment of the damaged place was conducted to remove the external oxidized surface (Figure 5). This phase was performed in such way to obtain the best access to whole crack by the welder during the welding process (Figure 6). It was found during mechanical treatment that the bell’s structure is very porous, especially the external surface. This worsened the welding process. The welding process was conducted with the use of an oxyacetylene torch. During this process the bell edges were melted with the welding rods made earlier. Welding was conducted with the use of the “up method”. Better efficiency of welding and very good penetration of the whole thickness of the connected parts were obtained thanks to this method. It was possible to perform the weld with a single torch cut due to this method.

**Figure 7:**
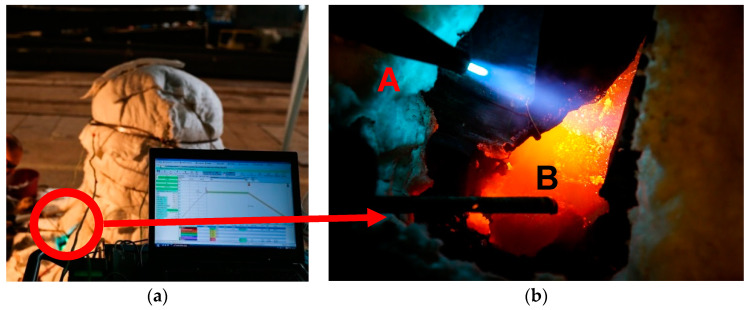
Welding process (**a**) the bell protected by fibro isolation; red circle–the place of welding (**b**) close–up of the place of welding. A—acetylene torch, B—bronze rod.

It was important to heat and keep the bell at the proper temperature during the welding process. This temperature was obtained by using heating mats and aluminosilicate fiber isolation. The whole process of heating was under the control of and recorded by a computer program (Figure 7);the heating rate was ~10 °C/h. The time of cooling after performing the weld was longer than the heating time, and the cooling rate was ~7 °C/h. Thus slow cooling was conducted to avoid stresses, which could have caused the bell to crack again. 

**Figure 8:**
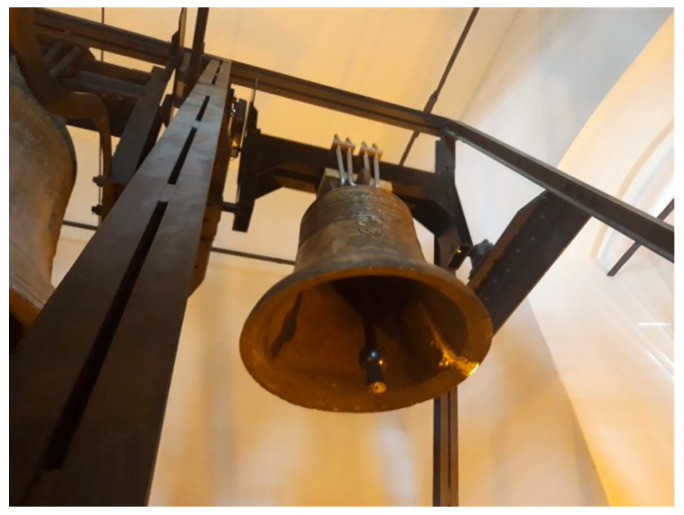
Welded bell attached to the tower.

After the cooling process, the place of welding was ground (Figure 8) and the sound of bell was examined.

## 4. Sound Analysis of the Bell

In 2013, before the crack, the bell’s sound was analyzed. After welding and the thermal stabilization process, this analysis was repeated. The sound of the bell, a G#4 note, did not worsen or even improve as a result of the negligible reduction of the main aliquots frequency. 

The target frequencies for the first partials are in the ratios 0.5:1.0:1.2:1.5:2.0 and these needed to be quite closely matched. The first aliquot, called the hum, is not prominent, and the perceived pitch is usually that of the second aliquot, called the prime, perhaps because it is reinforced by the harmonically-related aliquots with relative frequencies 2, 3, and 4. The tone of the bell is complex, however, particularly because of the presence of the minor-third (From Old French tierce, from Latin tertia) interval of 1.2 [10]. 

The lower (tone lower than the prime about the octave) and upper (tone higher than the prime about the octave) octaves with prime, tierce and quint were found to be beautifully harmonious after the repair. There was no distortionary vibration and the bell sustained its note for a long time.

**Figure 9:**
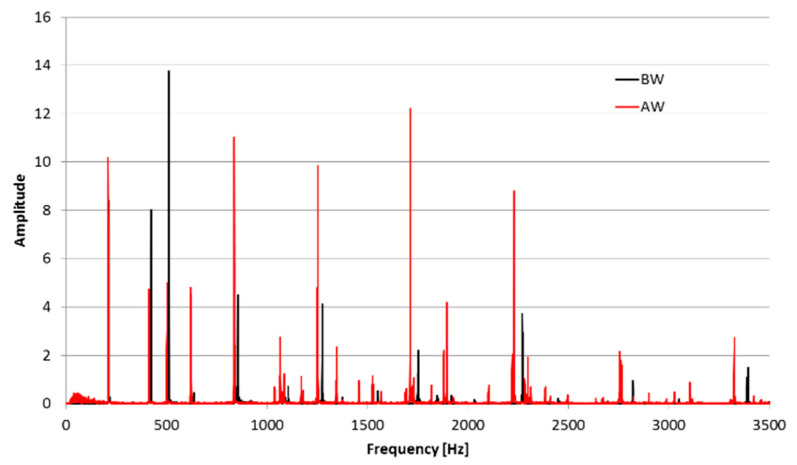
The spectrum of the St. Maryan bell’s sound, before (BW) and after (AW) welding.

**Figure 10:**
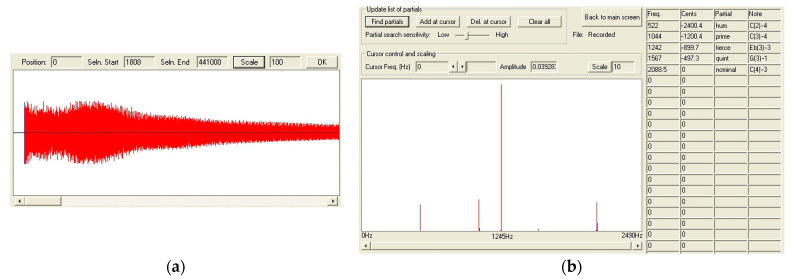
The analysis of sound wave emitted by bell C3 in program Wavanal; (**a**) the shape of recorded wave, (**b**) its spectrum.

The frequency spectrums of the Maryan bell before and after the crack are presented in Figure 9. The units of the amplitude in the figure are arbitrary; they are measured as voltages from a microphone, i.e., sound pressure levels on a linear scale. The amplitude of the spectrum is described in decibel scale. The program Wavanal [11] was used to analyze the sound of bell examined to determine the spectrum of emitted sound waves. This program was developed by W. A. Hibbert [12] for the sound analysis of bells to determine the influence of side tones on the height of the perceived strike tone (pitch tone). The possibility of fast and precise determination of their frequency was crucial, and the Wavanal program enabled it. This program allows a Fourier transform of sound waves directly recorded by a microphone joined to a computer or saved in a sound file recorded with other devices to be performed (Figure 10a,b). This program has received recognition among many bell makers as a great device for the evaluation of a bell’s sound and the process of tuning it up.

**Figure 11:**
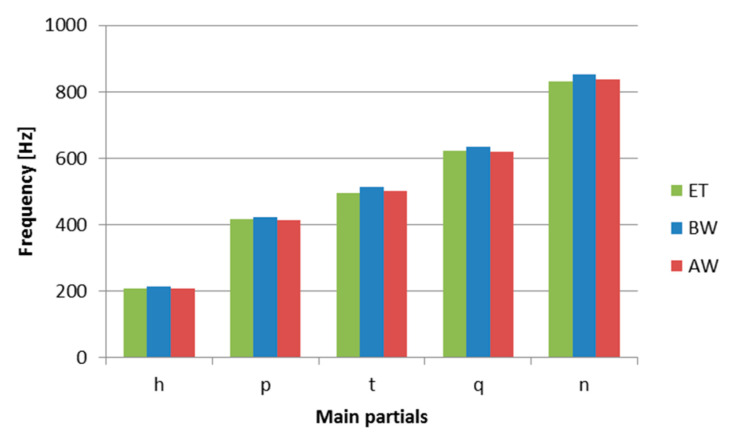
The main partials of the St. Maryan bell’s sound frequencies, before (BW), after (AW) welding and for musical note G#4 according to the equal temperament scale (ET).

Determined by the Wavanal program, values of frequency of basic side tones (aliquots): the lower octave (hum), prime (fundamental), minor tierce, quint, and upper octave (nominal) of the bells examined are presented in Table 3 and as a diagram in Figure 11.

## 5. Conclusions

Based on the experience gained during the work and research carried out on the Maryan bell, the following conclusions can be drawn:

1. If the welding process is carried out with the correct parameters, especially with thermal ones, and with monitoring and control of the heating and cooling rates, the repair even of contaminated high tin bronze bells is possible;
2. After repair by welding, the bell has a “better” sound, and this is probably due to a kind of heat treatment performed, including a cycle of slow heating and cooling which improves the properties of the material; and
3. The most highly recommended technique for repairing bells is gas welding, due to the relatively low temperature in the bonding area and the efficiency of the process.

## Figures and Tables

**Table 1:** Chemical composition of the Maryan bell (wt. %).

Sn	Pb	Sb	Zn	Fe	Ni	Ag	Cu
15.2	2.84	2.69	0.35	0.03	0.41	0.15	bal.

**Table 2:** Chemical composition in particular examined places presented in Figure 3.

Figure 3a			Figure 3b		
Number	Element	Atomic	Number	Element	Atomic
	Symbol	Concentration		Symbol	Concentration
1	Cu	21.43	1	C	75.57
	C	70.37		Zn	12.53
	Sn	1.77		Cu	3.56
	O	6.44		S	5.12
				O	3.22
2	Cu	28.54	2	Cu	17.76
	Sn	6.34		C	75.99
	C	57.87		S	3.79
	O	7.25		O	2.47
3	Cu	24.57	3	Pb	9.95
	C	64.84		C	64.53
	S	6.57		Cu	7.85
	O	3.84		O	17
	Sb	0.19		Sn	0.68
4	Cu	25.7			
	C	63.68			
	S	7.44			
5	Zn	24.46			
	C	57.29			
	S	13.12			
	Cu	2.88			
	O	2.25			

**Table 3:** Main partials of the St. Maryan bell’s sound frequencies before and after welding in comparison to harmonic tones for the musical note G#4.

Partials Tone	BW (Hz)	AW (Hz)	G#4 (Hz) (ET)
Hum	212.5	208.5	207.6
Prime	422	413	415.3
Tierce	512	502.5	493.8
Quint	635	619	622.3
Nominal	853.5	836	830.6

## Data Availability

The data presented in this study are available in article.
